# La Grippe—Its Symptomatology

**Published:** 1917-01

**Authors:** J. H. Wysong

**Affiliations:** Hico, Texas


### La Grippe—Its Symptomatology. Etiology and Treatment.

BY J. H. WYSONG, M. D., PH. D., HICO, TEXAS.

Influenza dates back at least to the twelfth century, when it ravaged Italy.

While occasional appearances have been noticed since its first visit to the New England colonies, in 1626, its severe characteristics were scarcely recognized in the United States until it became epidemic in 1889, after traveling westward from Petrograd, Berlin and London.

*Symptomatology.*—In the majority of cases the disease is ushered in by a rigor, or chilly sensations, violent headache, marked pain in the orbits, aching in the back, loins, and muscles of the limbs, hacking cough and a temperature ranging from 102° F. to 105° F. Extreme prostration is characteristic, develops early, and is out of proportion to the duration and severity of the catarrhal manifestations. Dyspnea is often a conspicuous symptom; sweating is at times troublesome and may lead to fatal collapse.

*Etiology.*—While Pfeiffer's bacillus influenzae has been honored since 1892 as the cause of grippe, the more modern studies serve to raise a question as to whether this honor should not be divided among several organisms.

In many localities during last year's great epidemic, the bacillus influenzae and micrococcus catarrhalis were not found, but in their absence pneumococci of various strains and hemolytic streptococci. The organisms mentioned are all foes of men, but which is the chief enemy, in any given epidemic, deserves further investigation by bacteriologists and clinicians throughout the land.

*Treatment.*—Its ubiquity, persistence from year to year in the same locality, severe onslaught, possible complications, and tendency to aggravate existing conditions and develop latent tendencies, render influenza one of the most-to-be-dreaded maladies that can afflict humanity. Its savage qualities should ever be borne in mind by the conscientious physician as he passes the threshold of every grippal victim, and would he not be more fitly armed for the contest should his heart be filled with the beautiful sentiment of Abraham Lincoln's favorite poem: "Oh, why should the spirit of mortal be proud?"

The laity very often fail to recognize the serious import of grippe and suffer many otherwise avoidable complications as a result. It is, therefore, the duty of the medical attendant to advise his uninformed patients and impress upon them the fact that seemingly mild cases are as likely to be fraught with severe complications and followed by as undesirable sequelae as those of the severest type.

Every patient suffering of la grippe should at once be put to bed and kept there, regardless of the mildness of his symptoms, until the fever has abated. He should be isolated from other members of the family, more particularly the very young and the old, in whom serious complications are likely to arise during the course of the disease. He should be housed in a large, well ventilated, sunlit room with only one attendant when practicable.

With these sanitary comforts provided for, a careful physical examination of the patient should follow. This should especially reveal the physical integrity of the three anatomic systems commonly involved in grippal infection. Of equal importance is the individualization of the patient; his hereditary tendencies, personal history and present illness.

In the case of the overworked general practitioner, the investigation of details of only academic importance are out of the question; but, on the other hand, hasty generalizations leading to illogical conclusions and ill-advised plans of treatment are to be deprecated.

In the beginning of an attack of grippe the patient's bowels should be freely opened with a mild saline cathartic or castor oil. Since there is no specific for this disease, the subsequent treatment is symptomatic.

For the relief of headache, muscular pain and general discomfort, some salicylic preparation, or coal-tar product may be judiciously administered. Their use should not be continued when the patient can comfortably endure this ordeal without them. Aspirin is a favorite remedy, but often occasions profuse sweating, much to the discomfort of the patient, and in this way predisposes to collapse. It is best given in moderate dosage in combination with citrated caffein. Codeine may be added when pain is unusually severe. It is needless to multiply remedies, every physician of experience has his favorite analgesic and anodyne for such cases, and should stand by them, bearing in mind that these agents are all depressants and should be given cautiously to grippal patients, more especially to the very young and old and the debilitated from any cause.

For the dry, persistent cough, apomorphine in small repeated doses is a capital remedy. This promptly liquefies the sputum and thus relieves. For substernal pain, morphine hydrochloride may be added.

After free expectoration is established stimulating, non-nauseating expectorants are indicated. For the relief of the cough and bronchorrhea of old age, dilute nitric acid, tincture nux vomica and spirit of chloroform in a suitable menstruum is the writer's favorite prescription.

When the initial painful symptoms are under control the efforts of the physician should be directed toward the support of the patient and the elimination of toxins.

The dietetic support is possible only after the first two or three days of the disease and should consist of nourishing food; milk, cereals, the white of eggs, with lemonade or water in large quantities to aid in eliminating poisons. To stimulate the secernent functions of the liver, kidneys, and intestinal canal, small doses of calomel, cautiously repeated, have, in the writer's experience, surpassed all other remedies, both as to rapidity and efficiency of action. At the same time moderate doses of quinine, with camphor if patient be nervous, find a useful application and materially aid in establishing convalescence.

During a normal convalescence there is no more efficient tonic than the elixir of iron, quinine, and strychnine, with an essence of pepsin, should a debilitated condition of the stomach persist.

Normally convalescence from la groppe is slow, and relapses frequent.

The patient should be warned against overexertion, his heart should be closely watched, an irregular or feeble pulse should be sustained by small doses of strychnine alone or with arsenic.

The diet should be gradually increased until a well balanced ration can be indulged without discomfort. Plenty of rest and fresh air should also be advised during convalescence.

In conclusion, the treatment of complications should be that of the complicating disease modified by the requirements of the initial or primary infection.

Profound prostration is a characteristic of grippe and should be considered in the management of all complicating diseases, and adequate support, both medicinal and dietetic, provided.

Even in young persons, during the first or second week of the attack, the signs and symptoms of the so-called "influenzal heart" appear. These are not stealthy in their approach as they appear in diphtheria, but usually obtrusive, and alike distressing to the patient and baffling to the physician.

Among those of middle and advanced life, especially the latter who bear the impress of arterial sclerosis and myocardial degeneration, still more obtrusive is the symptomatology. An irregular, rapid pulse, dyspnea, pallor, cyanosis and restlessness, point unerringly to acute poisoning of the cardiac musculature and dilating chambers.

In the presence of this symptom-complex the skill of the clinician is often severely taxed. Ultimately his solution of the problem will depend not on set formulae and rule-of-thumb methods of treatment, but on his daily management of the patient, adapting his treatment to the varying conditions present—now stimulating, now sedating, now sustaining.

Fleeting and unsubstantial are all the aids drawn from the pharmacopeia in such crises: digitalis, strophanthus, strychnine, caffein, ether, ammonia, camphor have their advocates. The first mentioned is the most disappointing in acute cardiac toxemia with high fever, and the most harmful perhaps in old cases with cardiovascular degenerations. Grave infection and pyrexia do not counteract the effect of strophanthus as they do digitalis, and again strophanthus may turn the tide in favor of the patient even in the presence of senile changes. Strychnine acts as a general vital excitant, but is not dependable as a cardiovascular stimulant. Caffein is an excellent stimulant, but whether really beneficial to the heart in these cases remains to be demonstrated.

Ammonia, ether and camphor are to be looked upon as temporary restoratives rather than permanent supports, still they have a place as emergency circulatory stimulants.

After all, drugs are of little avail in the treatment of acute cardiac infections. More important still is to conserve the strength of the heart by lessening its work in every possible way.

Freedom from noise, position in bed, digestible food, and general bodily comfort are factors of success to be reckoned with in all cases.

Finally, when convalescence has begun, all sources of excitement calculated to disturb the heart's action should be avoided—and graduated exercise permitted only when the dilated heart has recovered.

